# Fuzziness and Heterogeneity of Benthic Metacommunities in a Complex Transitional System

**DOI:** 10.1371/journal.pone.0052395

**Published:** 2012-12-21

**Authors:** Vinko Bandelj, Cosimo Solidoro, Daniele Curiel, Gianpiero Cossarini, Donata Melaku Canu, Andrea Rismondo

**Affiliations:** 1 OGS, Istituto Nazionale di Oceanografia e di Geofisica Sperimentale, Sgonico, Italy; 2 SELC, Venezia, Italy; National Institute of Water & Atmospheric Research, New Zealand

## Abstract

We propose an extension to the metacommunity (MC) concept and a novel operational methodology that has the potential to refine the analysis of MC structure at different hierarchical levels. We show that assemblages of species can also be seen as assemblages of abstract subregional habitat-related metacommunities (habMCs). This intrinsically fuzzy concept recognizes the existence of habMCs that are typically associated with given habitats, while allowing for the mixing and superposition of different habMCs in all sites and for boundaries among subregions that are neither spatially sharp nor temporally constant. The combination of fuzzy clustering and direct gradient analysis permits us to 1) objectively identify the number of habMCs that are present in a region as well as their spatial distributions and relative weights at different sites; 2) associate different subregions with different biological communities; and 3) quantitatively assess the affinities between habMCs and physical, morphological, biogeochemical, and environmental properties, thereby enabling an analysis of the roles and relative importance of various environmental parameters in shaping the spatial structure of a metacommunity. This concept and methodology offer the possibility of integrating the continuum and community unit concepts and of developing the concept of a habMC ecological niche. This approach also facilitates the practical application of the MC concept, which are not currently in common use. Applying these methods to macrophytobenthic and macrozoobenthic hard-substrate assemblages in the Venetian Lagoon, we identified a hierarchical organization of macrobenthic communities that associated different habMCs with different habitats. Our results demonstrate that different reference terms should be applied to different subregions to assess the ecological status of a waterbody and show that a combination of several environmental parameters describes the spatial heterogeneity of benthic communities much better than any single property can. Our results also emphasize the importance of considering heterogeneity and fuzziness when working in natural systems.

## Introduction

Spatial heterogeneity in geomorphologic, hydrodynamic and biogeochemical properties, as well as in the composition and structure of biological communities, are common features of coastal and estuarine systems [1], [2]. In fact, the perception that in large and complex systems the different subareas characterized by different abiotic properties (habitats) are dominated by different communities is so common among scientists that it has become embedded in marine conservation directives (e.g., the Water Framework Directive). These directives require that the evaluation of the ecological status of a waterbody be conducted by comparing different waterbodies against different reference systems, depending on habitat type.

However, the task of identifying such different biological communities, describing their characteristics and relating them to environmental gradients or ecological processes is not straightforward. There are difficulties in operationally defining a community and its spatial boundaries and associating quantitative variables to communities’ spatial distribution (e.g., what is the ‘abundance’ of a community?). In fact, although it is relatively straightforward to explore relationships between species distribution and environmental factors [3], [4], exploring and modeling the quantitative relationships between environmental variables and community spatial structure is much more challenging.

The metacommunity (MC) concept offers a useful framework to explore and understand the spatial heterogeneity of biological assemblages and the underlying ecological relationships. By defining a local community as the assemblage of species observed in a site and the MC as the set of local communities linked by the dispersal of multiple and potentially interacting species [5], [6], the MC concept posits that the observed composition of a local community results from the superposition of local (e.g., species interaction, species-environment relationships) and regional (e.g., migration, dispersal) factors, explicitly recognizing that communities have a spatial structure.

**Figure 1:**
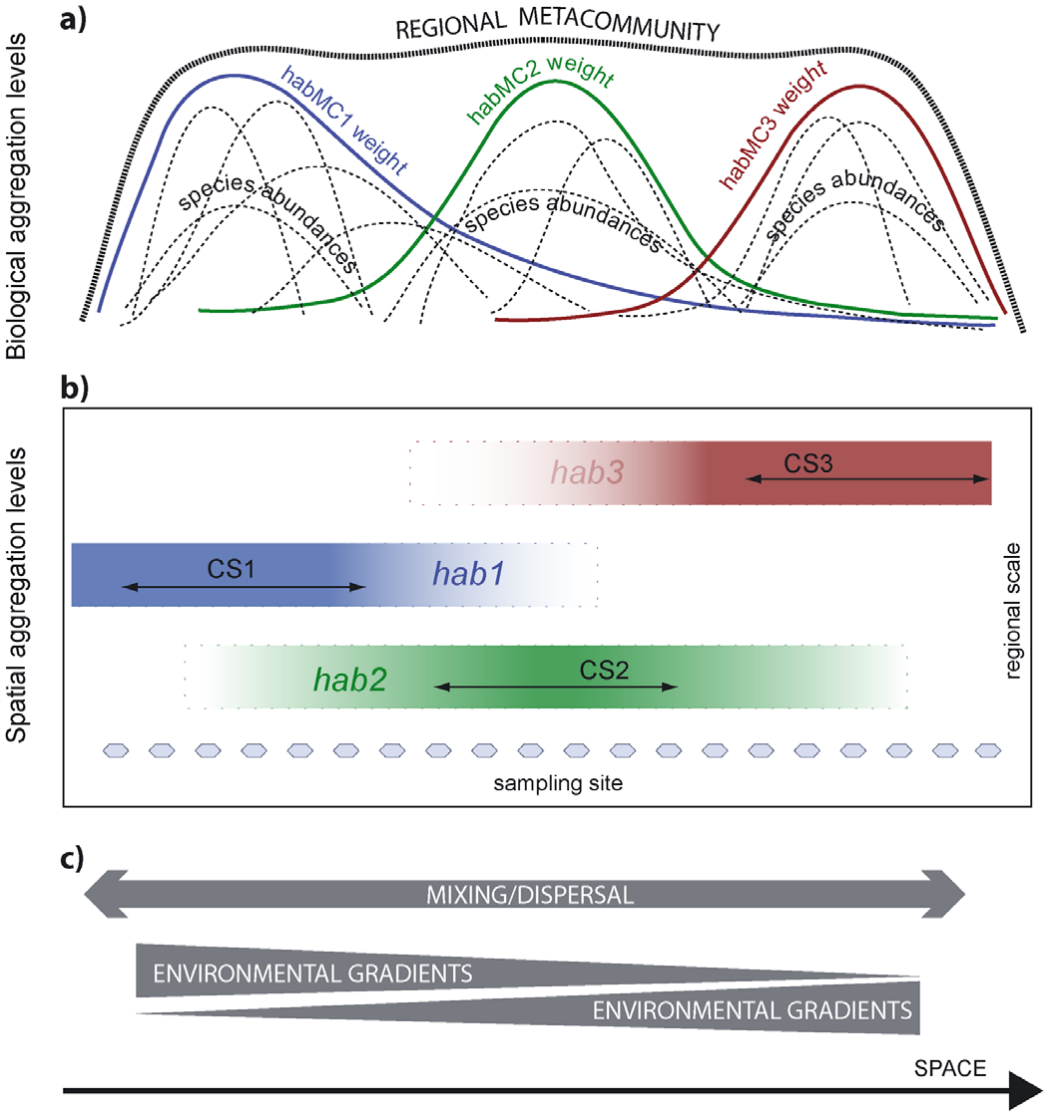
Conceptual diagram of the hierarchical spatial organization of macrobenthic communities. A) Different levels of biological aggregation (regional metacommunity, habitat metacommunity, species). B) Spatial scales (region, habitat, site). C) Environmental gradients and mixing/dispersal processes.

When considering complex systems characterized by significant habitat heterogeneity, the MC concept can be extended by adding an intermediate level of aggregation. The system can be subdivided into different and relatively fewer heterogeneous subregions still connected by dispersal processes, with each most suitable to (and preferentially hosting) a subregional abstract metacommunity typical of that habitat type (hereafter referred to as ‘habitat metacommunities,’ habMC). In this conceptual model, there are three different spatial levels (the region, subregion, and site) and three different biological aggregation levels (the metacommunity, subregional metacommunity, and local community). Each site hosts a local community that can be seen as both an assemblage of species and the overlap of different, interacting habMCs. This extension therefore recognizes the existence of habMCs, typically associated with given habitats, while allowing for the mixing and superposition of different habMCs to occur in all sites and for boundaries among subregions that are neither spatially sharp nor constant in time (Fig. 1). This extension provides a convenient framework for the analysis of community–environment relationships and links MCs directly to the application of conservation directives.

The possibility that different habMCs can occur together, with each assigned a different weight or value reflecting its abundance in defining the composition of an assemblage in a given site, provides a useful conceptual framework for associating a continuous number, i.e., the relative weight, with each habMC in a given site. However, it remains challenging to determine how to operationally define the number of habMCs present in a region, their spatial distributions, and their relative weights at different sites.

Here, we used a simple methodology based on the combination of fuzzy clustering (FKM, [7]) and direct gradient analysis (RDA, [8]) to demonstrate how this methodology can be applied to meet this challenge and to clarify the role of environmental sorting in highly connected areas characterized by a marked gradient in environmental variables.

The concept of fuzziness [9] allows non-probabilistic uncertainties to be addressed [10] in a wide range of fields, including pattern recognition, decision making, and classification [7], [11], and has been already applied to ecological studies [12]–[14]. FKM is a generic case of the iterative relocation classification algorithm k-means; it does not assign each sample to a single class but, rather, to all classes through assigning different membership grades. These grades indicate the affinity of a sample to each cluster or the extent to which one sample exhibits the traits of that cluster. This method thus appears to be suitable for biological applications because fuzzy classifications can reproduce the inherent fuzziness of biological spatial structuring observed in the real world more realistically than ‘standard’ clustering methods, in which classes are sharply defined, discontinuities may be artificially introduced, and intermediate objects are forced into one of the classes.

This application of fuzzy clustering objectively identifies the number of habMCs that summarizes the variability in a regional MC, and the extent to which each site exhibits the traits of each habMC (i.e., its membership grades). Because they are continuous variables, membership grades can be used as direct inputs in a gradient analysis without the artificial coding required by other techniques for dealing with qualitative variables (e.g., dummy variables, [15]). The results of the gradient analysis indicate the role and relative importance of environmental parameters in shaping the spatial structure of each abstract habMC and, therefore, of the regional metacommunity as a whole.

In our case study, we analyzed the hard-substrate macrobenthic populations in the Venetian Lagoon. We chose these organisms because they are clearly linked to a site and because they are commonly considered to be good indicators of (and therefore sensitive to) environmental conditions. We considered both phyto- and zoobenthic components to encompass a broader range of species interactions and in consideration of the fact that these components compete with each other for space. The Venetian Lagoon is an appropriate case study because, due to a long history of intensive human use, it has a large number of submerged, artificial hard substrates (e.g., urban embankments and wooden navigation pylons), which are evenly distributed over a wide range of different lagoon habitats and have undergone a process of naturalization after a long period of submersion. Furthermore, despite strong water mixing, and therefore high connectivity between subareas, strong gradients in the biogeochemical, morphological and hydrodynamic properties of the water exist, which enable investigations of the importance of local environmental conditions, migration and regional dispersal factors in shaping MC structure. Habitat heterogeneity was considered in terms of hydrodynamic properties, 10 water quality parameters, and sediment composition data.

Our results 1) illustrate a novel approach for exploring the relationships between metacommunity structure and habitat heterogeneity and demonstrate potential applications of the metacommunity concept and the validity of those applications, 2) demonstrate the utility of fuzzy clustering in the analysis of complex natural systems, 3) clarify the role of different environmental parameters in shaping the spatial distribution of benthic communities, and 4) provide evidence for the role of environmental sorting in spatial dispersal processes even in a highly connected environment.

## Materials and Methods

### Ethics Statement

No specific permits were required for the described field studies because the study was funded and commissioned by the Magistrato alle Acque di Venezia – the Water Venice Authority, which regulates all activities in the lagoon. The sampled areas are not privately owned, nor are they protected, and the field studies did not involve endangered or protected species.

### Study Site

**Figure 2:**
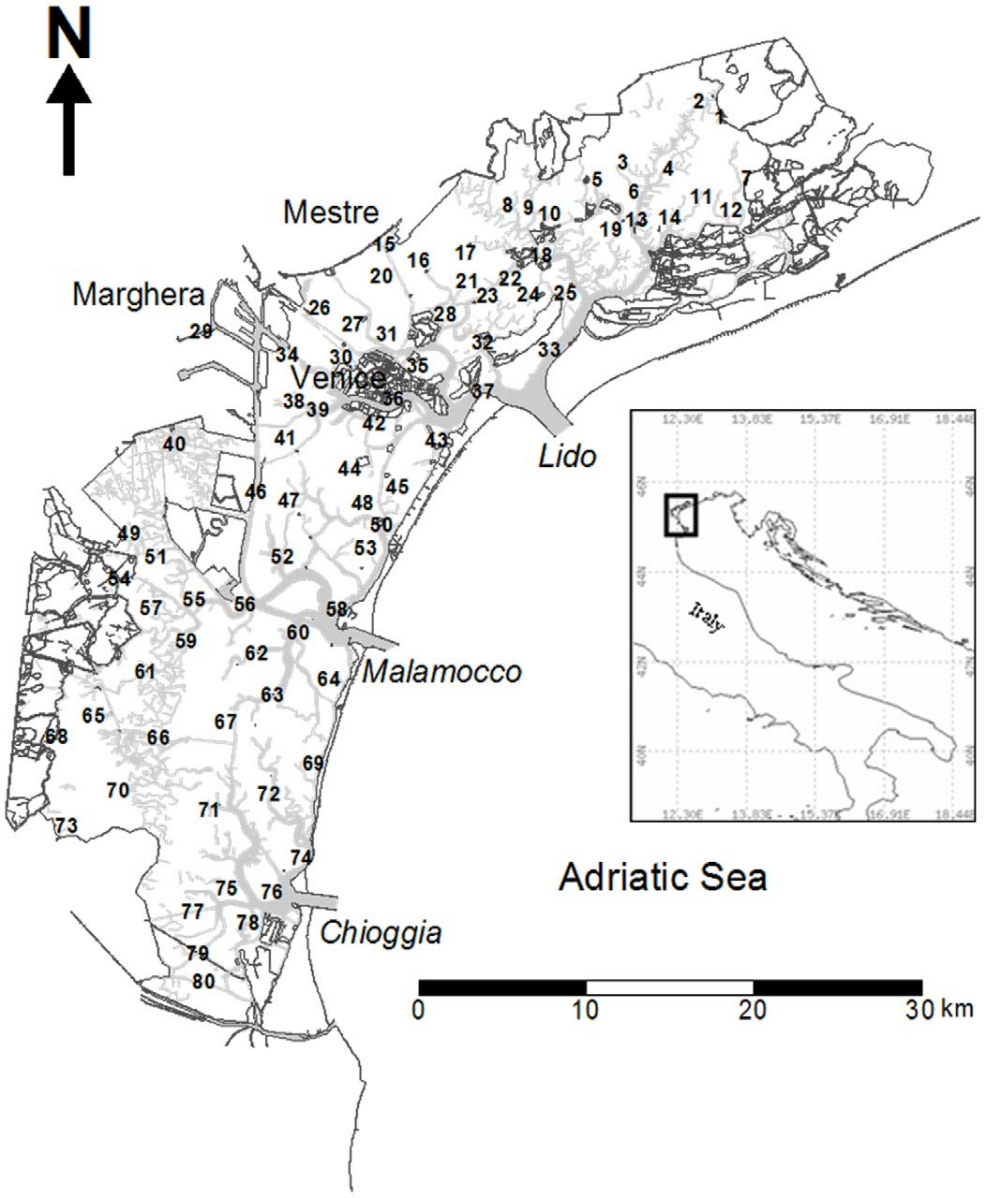
The Venetian Lagoon and the macrobenthos sampling sites.

The Venetian Lagoon is a microtidal lagoon in the northern part of the Mediterranean Sea along the northeastern coast of Italy (Fig. 2). It covers an area of approximately 550 km^2^, with approximately 150 km^2^ of this area closed to the sea and devoted to extensive aquaculture. Another 50 km^2^ of this area is occupied by islands. The lagoon is a complex, open system in which different anthropogenic activities interact and coexist with natural forces [16] and where strong gradients in physical-chemical [17], morphological [18] and hydrological parameters [19], [20] and ecological communities [21] are commonly observed. A complex network of channels originates from the three inlets that connect the lagoon to the Adriatic Sea, delimiting and connecting wetlands, tidal flats and islands. The average depth of the lagoon is approximately 1 m in shallow areas and up to 10 m in the main channels. A semidiurnal tidal regime sustains exchanges that reach 8000 m^3^s^−1^ and amounts to approximately 1/3 of the lagoon’s total volume per tidal cycle [22]. The actual renewal time for water in the lagoon varies from a few days for areas close to the inlets to up to 30 days for the inner areas [20]. There are 12 major tributaries that discharge an average of 35 m^3^s^−1^ of freshwater [23] into the lagoon annually. Total nutrient loads, including contributions from the river and urban and industrial sources, average approximately 4500 tN y^−1^ and 250 tP y^−1^ [24].

### Biological Variables

The macrophytobenthos and macrozoobenthos data were collected from 80 sites (Fig. 2) evenly distributed throughout the different habitats of the Venetian Lagoon (e.g., shallows, tidal flats, sea inlets, and channel side-shallows), with the exception of channels and industrial and urban areas (one site, number 29, was located inside an industrial channel). We sampled the following long-term, submerged artificial substrates: concrete substrates, where present (56 sites), and wooden (19 sites) and iron (5 sites) substrates otherwise.

The samples were obtained by scratching an area of 2000 cm^2^ with a blade from 20 cm above the mean sea level to 80 cm below it. Sampled organisms were separated and identified using a microscope, and the total surface area covered by each species was recorded in cm^2^ [25]. We used cover data rather than counts or biomass because this method prevented difficulties related to the identification of single specimens of colonial species; furthermore, the macrozoobenthic biomass is usually much higher than that of the macrophytobenthos, which would have then been underweighted in a count- or biomass-based analysis.

### Hydrodynamic Parameters

A finite-element hydrodynamic model developed and validated for the Venetian Lagoon [19], [26] was used to compute the spatial distribution of hydrodynamic properties (Table 1); the root mean square velocities, or RMS_V, were used as an indicator of the kinetic energy of the water circulating in the lagoon, and a measure of confinement (T_RES) was used as an indicator of transport time scales [27].

**Table 1:** Principal statistics of the environmental parameters used in the study.

Variable	Units	Min	25 perc	Median	75 perc	Max	IQR	Mean	StdDev
**TRES_M**	days	1.55	5.6	8.33	10.46	28.79	4.86	8.23	3.94
**RMS_V**	m/s	0.02	0.09	0.11	0.16	0.54	0.07	0.13	0.08
**TEMP**	°C	16.44	16.83	17.07	17.35	18.43	0.53	17.18	0.46
**SAL**	psu	21.28	28.11	30.44	31.47	33.77	3.36	29.82	2.3
**CHLA**	µg/L	1.33	2.21	3.08	4.21	8.83	2	3.47	1.52
**NH4**	µg/L	63.07	91.36	107.08	124.1	207.75	32.74	109.16	27.3
**NOx**	µg/L	152.99	282.03	350.59	463.76	961.77	181.72	370.11	139.19
**PO4**	µg/L	1.66	3.5	6.23	10.5	23.99	7	7.72	5.49
**DOC**	mg/L	2.77	3.05	3.18	3.32	3.98	0.27	3.25	0.29
**DON**	µg/L	111.74	132.18	152.31	185.14	286.34	52.96	167.29	45
**DOP**	µg/L	7.73	10.42	11	12.18	15.62	1.77	11.49	1.82
**POC**	mg/L	0.4	0.52	0.64	0.81	1.17	0.29	0.7	0.21
**TSS**	mg/L	12.38	17	18.85	21.07	38.5	4.07	19.74	4.31
**CLAY**	%	2.69	5.19	6.77	10.21	20.88	5.02	7.92	4.1
**SILT**	%	28.84	58.21	68.49	75.15	80.93	16.94	64.77	13.59
**SAND**	%	7.46	14.82	22.68	35.4	68.14	20.59	27.31	16.02
**DEPTH**	cm	40	70	90	150	650	80	126.84	98.8
**SUBSTRATE**	Concrete	Iron	wood						

Min = minimum, 25 perc = 25^th^ percentile, 75 perc = 75^th^ percentile, Max = maximum, IQR = Inter-quartile range, StdDev = standard deviation. Substrate type was coded as three dummy variables: Concrete, Iron, Wood.

In the RMS_V computation, the model was forced with data from wind fields and water levels at inlets measured in 2004 by the Municipality of Venice. The discharge values of rivers were taken from [23]. The RMS_V was computed for each element of the grid domain as the root square of the depth of integrated water velocity at each point over the whole year of simulation.

We analyzed the indicators of transport time scales by computing the time required for each element of the domain to accumulate a given amount of a conservative tracer initially dispersed in the sea, which is equivalent to the time required for each element to replace a given fraction of its volume with marine water [27]. This measure is, by definition, a very good approximation of the confinement [28] of each domain element and can be considered a modification of the remnant function [29]. To generate a value representative of the whole year, the procedure was repeated 12 times, starting on the first day of each month and using corresponding real-world wind and water levels and then taking the average of these values. The model gives values for RMS_V and T_RES at approximately 5000 points. The spatial resolution varies between 10 and 100 m, depending on morphology.

### Water Quality Parameters

Water quality was described by a set of 10 parameters (Table 1). The parameters were sampled monthly during 2004 at 20 stations evenly distributed throughout the lagoon and were spatially interpolated over a 100 m×100 m sampling grid covering the whole lagoon. Following [30], we used the Inverse Distance to a Power gridding method as the smoothing interpolator, the square of the inverse distance between new and known points as the weights, a searching radius of 7000 m, a minimum number of 4 data points, and a smoothing parameter of 0.05. This methodology was preferred over more sophisticated kriging methods because it enabled us to compute the distance between points by taking the presence of physical barriers (e.g., islands) into account [30]. The density of sampling points (approximately 1 point per 18 km^2^) is lower than the densities of the other properties analyzed in this study but can still sufficiently describe the spatial distribution of water quality parameters given that this lagoon is a shallow system subjected to intense tidal mixing twice a day. In fact, the water quality monitoring program for the Venetian Lagoon originally included 31 sampling sites, but the number of stations was reduced to 20 sites after 3 years and then further reduced to only 15 sites without a significant loss of information [30].

### Morphological, Sediment and Substrate Properties

We collected sediment composition data for the percentages of clay, silt and sand in the uppermost 5 cm of the surface layer. Data were gathered in 2004 at 103 stations evenly distributed throughout the lagoon and were interpolated to the 80 hard-substrate sampling sites using the interpolation method described above (Table 1). The searching radius was reduced to 3000 m because the increased density of sampling points allowed for a more accurate interpolation.

At each sampling site, the water depth and type of substrate were recorded during sampling (Table 1).

### Statistical Methods

The dataset was analyzed first by performing fuzzy clustering on the data for macrobenthic taxa coverage (fuzzy k-means or FKM [7]) and then by applying a direct gradient analysis method (redundancy analysis or RDA [8]) to the results of the FKM.

The number of classes used was determined jointly to the fuzzy exponent Φ, which determines the amount of fuzziness, by optimizing the Normalized Classification Entropy and the Fuzziness Performance Index [31], [32]. We computed 70 alternative fuzzy groupings by increasing N from 2 to 8 by steps of 1 and by increasing Φ from 1.1 to 2 by steps of 0.1 for each N. FKM also computes a confusion index (CI) for each sample, and this index is a measure of the degree of overlap of classes. Because our dataset had a high positive skewness (the mean skewness of the 168 taxa was equal to 6.35) and numerous zero presences, the macrobenthos coverage data were transformed with the Hellinger transformation prior to clustering [33] to overcome the ‘double zero’ problem [15].

We defined core subregions (CS) related to each fuzzy cluster as those sites with a membership grade above the 90th percentile of the distribution of all membership grades. The biological description of the clusters was obtained by determining the most abundant species in the cluster centroids. Furthermore, we computed the Fuzzy Membership Values (FMV) [34] for each taxon. FMV was computed by normalizing the taxa composition of the cluster centroids [34]. Characteristic species of the CS sites were obtained using the IndVal method [35]. We considered indicators to be only those taxa with an IndVal >25% and with an IndVal significant after 499 permutations of the samples and the cluster memberships. The spatial visualizations of membership and fuzziness were obtained using the Inverse Distance to a Power gridding method as the smoothing interpolator, the square of the inverse distance between new and known points as the weights, a searching radius of 3500 m, a minimum of 3 data points, and a smoothing parameter of 0.02.

RDA is an ordination procedure similar to Principal Component Analysis, with the exception that its ordination axes are limited to the linear combinations of a given set of environmental variables. For the RDA, the effects of substrate type were first partialled out by defining concrete, wood and iron as covariables [36]. Then, the remaining 17 environmental parameters were used as explanatory variables, and their partial effects, which were conditioned to the variance explained by substrate type, were computed. Using a forward selection procedure, we selected a subset of significant environmental variables (parsimonious model) with the highest explanatory power for variances in benthic communities [37]. The significance of each conditional contribution was tested after 499 Monte Carlo permutations, and p<0.05 was considered to be the threshold for significance.

## Results

### Biological Data

We observed a total number of 168 different benthic organisms (Table S1), and 141 of these were identified at the species level, while the others were identified according to higher taxonomic ranks. There were 89 macrophytobenthos (49 Rhodophyta, 24 Chlorophyta, 15 Ochrophyta, and 1 Chrysophyta) and 79 macrozoobenthos (14 Mollusca, 13 Bryozoa, 12 Polychaeta, 11 Tunicata, 8 Crustacea, 7 Porifera, 7 Hydrozoa, 5 Echinodermata, 1 Anthozoa, and 1 Polyplacophora). The total cover of macrozoobenthos (87932 cm^2^) was almost three times that of macrophytobenthos (31249 cm^2^). The mean cover per site was 1490 cm^2^, with a maximum cover of 3419 cm^2^ at station 36 and a minimum of 172 cm^2^ at station 11. The mean number of species per site was 38, with a maximum of 67 at station 32 and a minimum of 13 at station 73.

### Environmental Parameters

**Figure 3:**
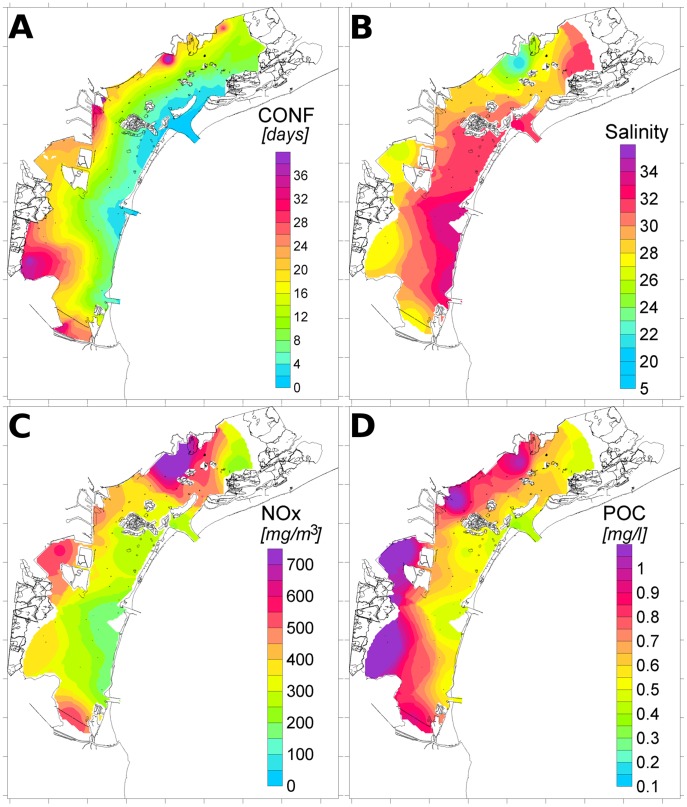
Spatial interpolation over the Venetian Lagoon of: confinement (A), salinity (B); nitrates (C); particulate organic carbon (D).

The statistical indices of the environmental parameters are given in Table 1. Spatial distributions over the whole lagoon are reported in Fig. 3 for T_RES, salinity, NOx, and particulate organic content (POC). The confinement is lower close to the lagoon inlets and increases landward in some areas of reduced exchange with the sea, especially in the southwestern part of the lagoon. The salinity is also higher close to the inlets and along the main channels. A clear freshwater signal is located in the northern part of the lagoon, close to the major river estuaries, where the highest concentrations of inorganic nutrients are also found. The areas between and south of Venice and Porto Marghera are rich in inorganic nutrients and organic matter due to wastewater discharges.

### Fuzzy Clustering

**Figure 4:**
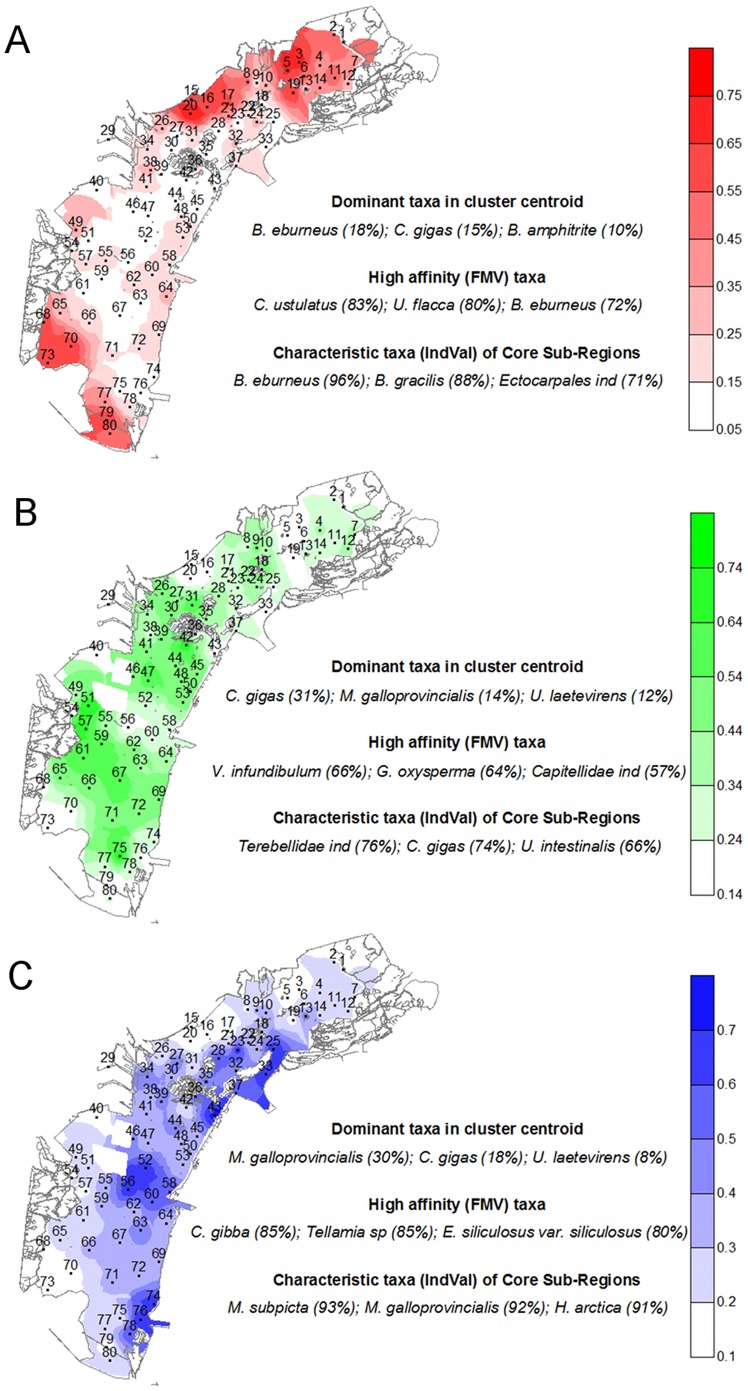
Spatial distribution of membership grades for the three habitat metacommunities habMC1 (A); habMC2 (B); habMC3 (C). Dominant and characteristic species are also indicated. All habMCs are present (with different relative weights) in most of the sites, indicating the importance of migration and dispersal processes through water mixing. Areas with high membership for a given habMC represent its CS. CSs do not overlap, indicating the importance of sorting by environmental gradients. Areas in which two or three habMCs have similar membership grades present mixed traits and are areas in which dispersal processes and environmental sorting are balanced.

The sites were clustered in three groups representing three habMCs (Fig. 4). The membership grades varied from 0.06 at site 51 to 0.73 at site 20. Several sampling sites were almost equally attributed to two clusters (sites 49 and 54 to clusters 1 and 2; sites 27, 34, 39, 44, 55, and 63 to clusters 2 and 3) or even to all three clusters (sites 10 and 38). Accordingly, the CI values ranged from 0.42 at site 20 to 0.98 at site 49 (highest fuzziness).

The CS for habMC1, CS1, included 10 sites (3, 5, 15, 16, 19, 20, 68, 70, 73, and 79), which are all in areas of reduced exchange in the northern and southern portions of the lagoon, far from the main channels. CS2 (sites 42, 51, 57, 59, 61, and 75) is located in the central and southern parts of the lagoon in shallow flats. CS3 included eight sites (23, 25, 33, 43, 52, 56, 74, and 76) and is located in the lagoon inlets and along the main channels.

### Gradient Analysis

After partialling out the effect of the substrate type, the RDA results (Table 2) indicated that salinity, sediment composition, hydrodynamics, and dissolved and particulate organic compounds have a similar statistical effect on the distribution of macrobenthic communities. As these parameters are statistically correlated (e.g., TRES_M shares half of its effect with salinity and more than half with DON, POC, and RMS_V), we built a parsimonious model starting with salinity and adding parameters one at a time insofar as they provided an independent and significant contribution to explaining the residual variance. Consequently, the total explained variance was lower than the sum of the individual effects of the included variables. The parsimonious model (Table 2) explained approximately 40% of the total variance in the cluster membership grades, and substrate type alone explained an additional 14% of the total variance.

**Table 2:** Marginal effects and partial effects in the RDA parsimonious model of environmental variables on FKM membership grades.

Marginal effects		Parsimonious model		
Variable	% Variance	Variable	% Variance	p
**SAL**	12%	SAL*	12%	0.002
**DOC**	10%	NOx*	6%	0.002
**SAND**	10%	POC*	6%	0.006
**POC**	10%	SAND*	5%	0.004
**DON**	9%	CLAY*	2%	0.038
**TRES_M**	9%	DON*	5%	0.004
**SILT**	9%	TRES_M*	3%	0.018
**RMS_V**	9%	**DEPTH**	2%	0.056
**CLAY**	7%	**RMS_V**	1%	0.098
**DOP**	6%	**CHLA**	2%	0.138
**NOx**	6%	**TSS**	0%	0.322
**DEPTH**	5%	**NH4**	1%	0.444
**CHLA**	4%	**TEMP**	0%	0.428
**TSS**	3%	**DOP**	1%	0.166
**TEMP**	2%	**SILT**	1%	0.742
**PO4**	1%	**PO4**	0%	0.862
**NH4**	0%	**DOC**	0%	0.522

Marginal effects were conditioned to substrate type, which explained 14% of total variance. Variables are ordered according to decreasing marginal and decreasing partial effects. Asterisks mark variables included in the parsimonious model. P = significance level of variables’ contribution in the parsimonious model.

**Figure 5:**
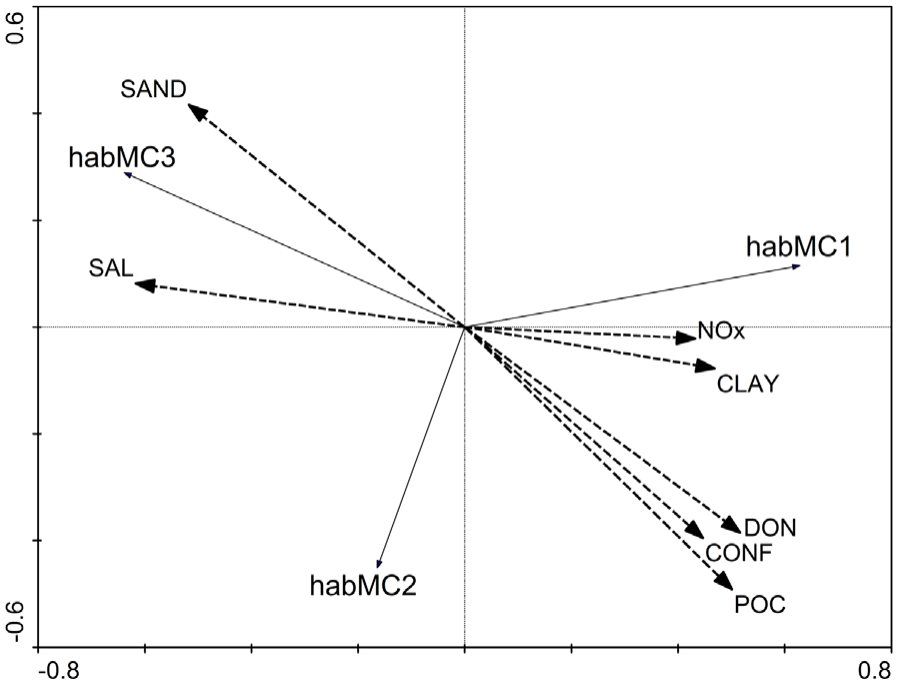
Biplot of the RDA parsimonious model explaining fuzzy memberships for the three habMCs.

The main gradient along which habMC1 and habMC3 are ordered (Fig. 5) can be described as a linear combination of decreasing salinity and sand content in the sediment and increasing clay content and nitrate concentration in the water. The second gradient, along which the ‘intermediate’ community is oriented, has high POC and DON concentrations, high TRES_M, and high sand content in the sediment. The parsimonious model explained 40.8%, 49.2% and 23.1% of the variance in the membership grades for habMC1, habMC3, habMC2, respectively. The fuzziness of habMC2 and the reduced characterization of its CS by indicator species are reflected in the reduced ability of the parsimonious model to predict the structure of this metacommunity.

### Biological Characterization of Clusters

Clusters can be biologically characterized using different indicators. The analysis of fuzzy cluster centroids indicated that the total sum of cover shows an increasing gradient from habMC1 (1114 cm^2^) to habMC2 (1568 cm^2^) and habMC3 (1758 cm^2^).

The three fuzzy cluster centroids were characterized by very similar populations (Table 3, Fig. 4). We found that the same eight species accounted for 75% of the total cover in habMC1 and habMC2 and that in habMC3, these eight species, along with two additional species, formed significant populations. Most species showed a clear gradient. For example, *Balanus eburneus* covers 18% of the total centroid’s area in habMC1 but only 3% and 2% in habMC2 and habMC3, respectively. *Crassostrea gigas*, *Ulva laetevirens* and *Ulva intestinalis* cover a much larger area of the centroid in habMC2 than in habMC1 and habMC3. The area covered by *Mytilus galloprovincialis* increases from habMC1 to habMC2 and habMC3.

**Table 3:** Percentage abundance for taxa in the centroids of the three habMCs.

Group	Taxon	habMC1	habMC2	habMC3
Anthozoa	Anthozoa ind	1.05%	0.62%	
Rhodophyta	*Antithamnion cruciatum*	0.56%		
Crustacea Cirripedia	*Balanus amphitrite*	9.53%	3.86%	3.58%
Crustacea Cirripedia	*Balanus eburneus*	18.22%	2.86%	1.94%
Bryozoa	*Bowerbankia gracilis*	1.19%		
Bryozoa	*Bugula neritina*		0.77%	
Rhodophyta	*Ceramium diaphanum*			0.65%
Tunicata	*colonial Botryllidae*		0.89%	0.79%
Mollusca Bivalvia	*Crassostrea gigas*	14.59%	30.59%	17.85%
Rhodophyta	*Dasya baillouviana*	0.67%		
Bryozoa	*Electra monostachys*	0.57%		
Phaeophyta	*Fucus virsoides*		0.57%	
Porifera	*Hymeniacidon perlevis*	5.24%	6.31%	6.02%
Mollusca Bivalvia	*Limnoperna securis*	1.85%	0.76%	
Mollusca Bivalvia	*Mytilus galloprovincialis*	7.55%	13.77%	29.50%
Rhodophyta	*Neosiphonia harveyi*			0.65%
Rhodophyta	*Polysiphonia denudata*	1.76%	1.91%	4.66%
Rhodophyta	*Polysiphonia elongata*	0.81%		
Rhodophyta	*Polysiphonia scopulorum*	1.83%	0.84%	0.84%
Rhodophyta	*Rhodymenia ardissonei*	0.76%	0.79%	0.99%
Tunicata	*Styela plicata*	0.99%	1.37%	0.84%
Porifera	*Tedania anhelans*	0.95%	1.33%	3.48%
Bryozoa	*Tricellaria inopinata*		0.80%	0.90%
Crustacea Amphipoda	tube-dwelling Amphipoda	9.46%	3.37%	3.43%
Chlorophyta	*Ulva flexuosa*	1.67%	0.98%	1.13%
Chlorophyta	*Ulva intestinalis*	3.93%	6.22%	3.86%
Chlorophyta	*Ulva laetevirens*	7.20%	11.82%	7.98%
Phaeophyta	*Undaria pinnatifida*			1.02%

Only the most abundant taxa are shown, i.e. those which cumulatively add up for >90% of the total abundance in the centroids representative of each of the three habMCs. Taxa are ordered by decreasing alphabetic order.

The species FMVs (Table 4, Fig. 4) show each taxon’s affinity for the three communities. Many of the taxa with high affinities are rare or present in low abundance and, as a result, are not identified in the analysis of centroids. In contrast, some of the most abundant species exhibit no clear preference for a community (e.g., *U. laetevirens*, *U. intestinalis*, *Hymeniacidon perlevis*).

**Table 4:** FMV for 15 taxa with highest affinities for each of the three habMCs.

habMC1				
Group	Taxon	habMC1	habMC2	habMC3
Rhodophyta	*Caulacanthus ustulatus*	82.76%	8.10%	9.14%
Chlorophyta	*Ulothrix flacca*	79.75%	9.35%	10.90%
Crustacea Cirripedia	*Balanus eburneus*	71.99%	15.92%	12.09%
Bryozoa	*Bowerbankia gracilis*	69.46%	16.91%	13.62%
Chlorophyta	*Cladophora vagabunda*	66.63%	19.48%	13.89%
Bryozoa	*Cryptosula pallasiana*	64.62%	21.58%	13.80%
Rhodophyta	*Chondria capillaris*	63.93%	16.85%	19.22%
Phaeophyta	*Ectocarpales ind*	61.94%	21.72%	16.33%
Bryozoa	*Electra monostachys*	60.53%	23.14%	16.33%
Rhodophyta	*Polysiphonia elongata*	58.33%	18.48%	23.19%
Rhodophyta	*Sahlingia subintegra*	55.52%	20.68%	23.80%
Rhodophyta	*Dasya baillouviana*	54.93%	22.23%	22.84%
Rhodophyta	*Callithamnion corymbosum*	53.65%	19.97%	26.37%
Rhodophyta	*Polysiphonia sp2*	52.30%	23.99%	23.71%
Phaeophyta	*Scytosiphon sp*	52.30%	23.99%	23.71%
**habMC2**				
**Group**	**Taxon**	**habMC1**	**habMC2**	**habMC3**
Polychaeta	*Vermiliopsis infundibulum*	11.95%	65.95%	22.10%
Chlorophyta	*Gayralia oxysperma*	17.31%	63.59%	19.10%
Polychaeta	*Capitellidae ind*	12.43%	57.44%	30.12%
Chlorophyta	*Blidingia minima*	25.07%	57.11%	17.82%
Phaeophyta	*Dictyota dichotoma var. intricata*	15.25%	56.68%	28.08%
Rhodophyta	*Ceramium ciliatum*	15.28%	56.60%	28.12%
Hydrozoa	*Hydrozoa ind*	12.73%	54.96%	32.30%
Bryozoa	*Scrupocellaria bertholettii*	12.10%	53.44%	34.45%
Chrysophyta	*Vaucheria submarina*	22.94%	52.88%	24.17%
Rhodophyta	*Peyssonnelia dubyi*	16.55%	52.85%	30.60%
Rhodophyta	*Gymnogongrus griffithsiae*	16.84%	52.49%	30.66%
Phaeophyta	*Fucus virsoides*	16.94%	52.39%	30.67%
Rhodophyta	*Centroceras clavulatum*	16.94%	52.39%	30.67%
Rhodophyta	*Melobesia membranacea*	16.94%	52.39%	30.67%
Echinodermata	*Asterina gibbosa*	13.01%	52.02%	34.97%
**habMC3**				
**Group**	**Taxon**	**habMC1**	**habMC2**	**habMC3**
Mollusca Bivalvia	*Corbula gibba*	2.55%	12.49%	84.96%
Chlorophyta	*Tellamia sp*	2.55%	12.49%	84.96%
Phaeophyta	*Ectocarpus siliculosus var. siliculosus*	5.63%	14.20%	80.16%
Rhodophyta	*Antithamnion piliferum*	7.66%	15.01%	77.33%
Rhodophyta	*Grateloupia filicina*	8.03%	15.23%	76.75%
Polychaeta	*Hydroides pseudouncinatus*	10.07%	13.30%	76.63%
Tunicata	*Ascidia mentula*	8.35%	15.87%	75.78%
Rhodophyta	*Grateloupia turuturu*	7.00%	20.29%	72.71%
Rhodophyta	*Radicilingua thysanorhizans*	6.60%	20.71%	72.69%
Echinodermata	*Ophiothrix fragilis*	10.41%	17.41%	72.18%
Echinodermata	*Ophiuroidea ind*	7.62%	20.23%	72.14%
Phaeophyta	*Dictyota dichotoma var. dichotoma*	13.62%	16.67%	69.71%
Porifera	*Tedania anhelans*	11.44%	22.46%	66.09%
Mollusca Bivalvia	*Anomia ephippium*	9.32%	25.21%	65.47%
Phaeophyta	*Pseudolithoderma adriaticum*	13.48%	21.15%	65.36%

Taxa are ordered by decreasing FMV in habMC1, habMC2, habMC3, FMV values are shown for each habMC.

Identification of the sites with the highest membership grades for each metacommunity made it possible to determine the abundance of taxa and their frequency in the CS. A biodiversity gradient was evident, with a low mean number of taxa present in the CS of habMC1 (26), a greater number in habMC2 (40), and the maximum number of taxa in habMC3 (52). IndVal values were calculated for the CS of the three clusters, and this allowed us to identify the characteristic species (Table 5, Fig. 4). The IndVal values were generally higher in habMC1 and habMC3 and lower in habMC2, confirming the stronger characterization of the former and the higher fuzziness of the latter. The number of indicator taxa was the highest in habMC3 and the lowest in habMC1, reflecting the general trend of biodiversity among the CS of the three metacommunities. HabMC1 was strongly characterized by taxa associated with areas of low water renewal and high organic loads. The 10 indicator taxa of habMC2 were associated with shallow and low-energy areas. The 19 indicator taxa of habMC3 were generally associated with areas of high energy and saline water.

**Table 5:** Indicator taxa of the three metacommunities’ CSs.

Group	Taxon	habMC1	habMC2	habMC3
Mollusca Bivalvia	*Anomia ephippium*			86.56%
Crustacea Cirripedia	*Balanus eburneus*	96.4%		
Chlorophyta	*Blidingia minima*		50%	
Bryozoa	*Bowerbankia gracilis*	87.86%		
Bryozoa	*Bugula stolonifera*			49.88%
Hydrozoa	Campanulariidae ind			62.5%
Rhodophyta	*Ceramium diaphanum*			77.34%
Mollusca Bivalvia	*Crassostrea gigas*		73.85%	
Phaeophyta	Ectocarpales ind	71.35%		
Bryozoa	*Electra monostachys*	62.88%		
Rhodophyta	*Erythrocladia irregularis*			72.66%
Chlorophyta	*Gayralia oxysperma*		65.6%	
Mollusca Bivalvia	*Hiatella arctica*			91.14%
Polychaeta	*Hydroides dianthus*		58%	
Porifera	*Hymeniacidon perlevis*			53.24%
Hydrozoa	*Kirchenpaueria halecioides*			65.11%
Mollusca Bivalvia	*Modiolarca subpicta*			92.63%
Mollusca Bivalvia	*Mytilaster lineatus*			62.81%
Mollusca Bivalvia	*Mytilus galloprovincialis*			91.95%
Rhodophyta	*Polysiphonia denudata*			72.82%
Rhodophyta	*Rhodymenia ardissonei*			71.44%
Bryozoa	*Schizoporella errata*			37.5%
Polychaeta	*Spirobranchus triqueter*			84.81%
Porifera	*Tedania anhelans*			85.92%
Polychaeta	Terebellidae ind		75.91%	
Bryozoa	*Tricellaria inopinata*			86.04%
Chlorophyta	*Ulva intestinalis*		65.96%	
Chlorophyta	*Ulvella lens*			45.82%
Chrysophyta	*Vaucheria submarina*		44.94%	

Only taxa with significative (p-level<0.05, 499 permutations) IndVal >25% are shown.

The increasing abundance and biodiversity gradients from confined areas to marine waters were in agreement with observations already reported for several Mediterranean lagoons [28], [38]. Our biological description of the benthic metacommunities in the Venetian Lagoon is coherent with results of previous studies [39]–[41], with some differences. These differences may have been caused by our reliance on an objective identification of the benthic communities and a posteriori derivation of their characteristics, rather than on expert judgments or single-species ecological literature.

### Sensitivity to Different Class Numbers

We performed additional analyses with a smaller (2) and larger (4) numbers of classes.

When using two classes, sites were subdivided between an ‘inner’ group, whose CS was roughly equivalent to the CS1 emerging from the three-group classification scheme, and an ‘outer’ group, whose CS included all other sites. In the two-group scheme, therefore, a major inner-outer gradient was still evident, but the subdivision between ‘intermediate’ and ‘marine’ habMCs was lost in spite of the fact that these subdivisions were quite different in terms of the species present. Thus, the two-group scheme was similar to but less accurate than the three-group scheme.

In the classification scheme with four clusters, there were two clusters that closely resembled the CS1 and CS3 of the three-group classification scheme. The third and fourth clusters showed almost exactly the same membership grades at all sampling stations. The stations with high membership grades for both of these clusters were those in the flat, low-depth areas, especially in the south-central part of the lagoon (CS2). Thus, it seems that in this case, the clusters began to be redundant, clearly indicating that the maximum “real” number of classes in the benthic assemblages of the Venetian Lagoon is 3.

## Discussion

The debate over the nature, structure and organization of living organisms has been ongoing for more than 100 years, with researchers either championing the merits of the individualistic, continuum and community-unit concepts or trying to integrate and reconcile these theories with one another [42]–[47]. We have contributed to this already rich theoretical debate by showing how the spatial structure of an MC (regional scale) can also be read as the superposition of a given number of distinct, coexisting and partially overlapping (abstract) habMCs (at the subregional scale), each of which is related to a particular habitat. A habMC is considered to be a unit (similar to the Clementsian approach), but the boundaries between habMCs need not be discrete, and the transition between subregions dominated by different habMCs can be smooth (as in the continuum individualistic approach), given that the relative weight of a habMC in a site can change continuously in space, similarly to the abundance of a species.

This extension of the MC concept and the related novel operational methodology proposed here create the possibility for a new level of interpretation/analysis in which the composition of the MC is also interpreted in terms of assemblages of habMCs rather than only in terms of assemblages of individual species. When exploring the impact of environmental variables on MCs, numerical analyses do not contrast environmental data and species abundances but, rather, environmental data and the relative weights of habMCs. Thus, our extension models the spatial distributions of habMCs, explores the affinities between habMCs and environmental variables and develops the concept of a habMC ecological niche that is analogous to the concept of a species’ ecological niche [48].

Our results highlight that the spatial distribution of the metacommunities is fuzzy and indicate that both environmental sorting and dispersal processes structure the benthic metacommunity and dominate it at different spatial scales. Our study site was subjected to strong tidal mixing and water exchange, causing different subareas to be highly connected. In the absence of strong environmental sorting, it would be reasonable to assume that the lagoon contained only one habMC. However, the objective identification of three distinct, although overlapping, habMCs supports the notion that – at the regional scale – environmental sorting exerts a stronger influence over community structure than dispersal does. The presence of mixed habMCs in all sites (no sampling site showed traits of only one cluster, while many showed highly mixed traits) and the similarities among habMCs (the three metacommunities shared the most abundant species in centroid composition) illustrates that dispersal processes do contribute to the structuring of the metacommunity. At smaller spatial scales (i.e., within a subregion) a single habMC predominates despite the presence of (relatively weak) environmental gradients, indicating that at these scales, dispersal processes prevail over environmental sorting.

Although all habMCs coexist, different habitat metacommunities are predominant (i.e., have greater membership grades, i.e., greater affinity) in different areas of the lagoon. HabMC1 showed high membership grades in the innermost shallow areas of the northern and southern lagoon basins and close to freshwater inputs (Fig. 4). HabMC2 was characteristic of low-depth flats of the central and southern basins. Areas with high membership for habMC3 were positioned near the three inlets and along the main channels rooted on the inlets. The interpretation of the three habMCs as ‘confined’ (habMC1), ‘intermediate’ (habMC2) and ‘marine’ (habMC3) was confirmed by the differences observed in the less abundant taxa in their centroids’ composition (Table 3), in the taxa with greatest affinity (FMV) (Table 4), and in the lists of indicator taxa of each CS (Table 5). Thus, our results clearly corroborate the existence of the hierarchical organization hypothesized in the introduction (Fig. 1): at the regional level, the macrobenthos in the Venetian Lagoon constitute a single metacommunity, effectively exchanging organisms between different subareas; at the same time, every habitat preferentially hosts its own habitat metacommunity, which differ in several indicator or sentinel species; the last step of this hierarchical organization is represented by single sites, where the actual composition can be described as a mixture of the habMCs present in the lagoon. Our results also emphasizes that fuzziness is a fundamental feature of empirical observations and exemplify how theories and methodologies that incorporate fuzzy concepts can be simple but effective in describing the fuzziness inherent in real-world metacommunities.

The scientific community recognizes that within large, heterogeneous systems, different subregions present different habitats and host different biological communities [1], [2]. This idea forms a basis of environmental paradigms [28] and has been embedded in marine conservation directives [49]. Although it is reasonable to assume that single species respond individually and at different spatial scales to environmental gradients, factors such as mutualism, symbiosis, and preferential dispersion routes can also result in discernible and repeated patterns of species association and, possibly, community structure. One possibility for analyzing the spatial structure of the metacommunity is to assume that different species respond to environmental conditions (possibly including interactions with other species) at an individual level [43], [44] and that communities are emergent properties. In fact, the analysis of species–environment relationships [45], [50] and the predictive modeling of species distributions [3], [51] have been popular and increasingly important topics. However, these methods often have limitations, mainly due to their lack of integration with ecological theory [51]. Among other shortcomings, species distribution models do not usually consider competition and other biotic interactions. Some attempts to incorporate these factors have been made [52], but the challenge persists due to the difficulty of identifying and understanding every type of biological interaction. Similar limitations apply to methodologies that consider a multivariate approach but still focus on species abundance, such as studies of neutral community theory [53], [54]. Furthermore, analyses performed on species abundances, such as RDA on actual species composition data, usually require the consideration of many principal components and a number of crowded ordination diagrams, which are difficult to interpret. The possibility of dealing with an entire habMC as an entity, and thereby implicitly overcoming these limitations, is therefore attractive, especially if it does not require that the continuum approach be rejected but, rather, as in our case, transposes that approach from the individual level to the community level [55]. We speculate that dealing with entire habMCs this way would be particularly useful in the analysis of communities including structuring organisms or other assemblages in which inter-species biotic interactions play an important role in defining the structure of the community.

The simple operational methodology presented here provides an objective identification of habMCs and quantitatively relates their spatial distributions to gradients in environmental variables. Other methodologies offer cluster interpretation through gradient analysis, such as Discriminant Analysis [57], but only for crisp classifications. Crisp clustering methods implicitly rely on the community-unit concept [42], [44] and do not account for continuum or hierarchical approaches. Furthermore, in these types of analysis, cluster type is a categorical variable, and prior to gradient analysis, it must be properly coded as a quantitative variable, usually through artificial procedures [15]. In contrast, fuzzy membership grades are continuous quantitative variables that can be directly used in direct gradient analysis. Their interpretation is straightforward: the higher the value of membership, the higher the affinity to a certain habMC. Thus, a canonical analysis of membership grades can indicate the strength and significance of statistical relationships between habMC traits and environmental parameters. Our choice to use the RDA methodology was informed by the specificity of our case study, but other gradient analysis methods (e.g., CCA, neural networks) can also be used for the interpretation of FKM membership grades. It should be noted, however, that this method, which involves treating the community as an entity, does not directly reveal the mechanisms underlying the observed patterns. but it does reveal possible associations between the taxa and both the environmental gradients and their dispersal abilities, from which hypotheses about mechanisms can be formed and tested.

A possible shortcoming of the proposed methodology is its ability to derive a biological characterization of the habMCs given that fuzzy classifications do not provide a crisp definition of these entities. However, the distribution of membership grades implicitly takes into account the single-species distributions on which the fuzzy clustering was performed. An obvious possibility is the use of cluster centroids to characterize the metacommunity composition in terms of the most abundant species. This approach can tend to overlook less abundant and less frequent species, such as rural, urban or sentinel species [47]. These species may be locally important or may be indicators of particular environmental conditions. The FMV approach [34] can partly overcome the problem of less abundant species. FMVs are an indication of the degree to which each taxon belongs to a certain fuzzy community; thus, they are the species’ counterpart of the sites’ membership grades. Because they rely on the relative contributions of species across the metacommunities, FMVs may illuminate the affinities of less abundant species, which are neglected when considering their percentage contribution inside each metacommunity. The primary benefit of FMVs is their ability to determine which habMC a single species is more closely associated with, rather than to describe metacommunities. The identification of the CS of each metacommunity, i.e., of sites with lower levels of uncertainty as to their community’s traits, permits the application of methods based not only on abundance but also on frequency, such as the IndVal applied in the present study. A sufficient number of sites should be included in each CS to avoid having to deal with singletons or the presence of sporadic species. Thus, even if FKM clusters are fuzzy and it is less straightforward to derive a biological characterization than with crisp clusters, our results demonstrate how several methodologies (the analysis of cluster centroids, FMVs, IndVal of CS) can be successfully used to this effect.

The exploration of community–environment relationships is also relevant to applied research and has been the subject of intensive study. The fuzzy habMC concept, by enabling the quantitative exploration of metacommunity–environment relationships, also offers novel possibilities to test theories on this topic and provides new insights and new evidence for existing paradigms. Thus, this approach contributes to the reconciliation of theoretical and empirical studies. For example, in brackish water systems, alternative paradigms have been proposed that classify bodies of water based on their salinity [57] or confinement [28], but different combinations of parameters (e.g., sediment type, water energy, temperature, and water quality) can also be used to do this [58], [59]. Our methodology enabled us to statistically assess the direct effects of 20 environmental variables and their linear combinations on benthic communities. Additional parameters, possibly related to human impacts (navigation, fishery, clam harvesting, industrial pollutants, etc.), could be considered to account for the fraction of unexplained variance [60]–[62]. However, in contrast to the theories noted above, our results indicate that no single parameter can be used as the ‘ultimate’ parameter shaping the lagoon’s zonation, and we found that a combination of several environmental parameters describes the spatial heterogeneity of benthic communities much better than any single property.

Similarly, the existence of distinct habMCs within a waterbody underlines the need to carefully choose the reference terms used to assess the ecological status of a waterbody [*sensu* the EU Water Framework Directive] and supports the idea that different reference terms should be applied to different subregions. This conclusion also stresses that heterogeneity is inherent in complex systems [1], [29], [63] and is a component of the total biodiversity that should be preserved.

## Supporting Information

Table S1 Macrobenthic taxa identified on hard-bottoms in the Lagoon of Venice in 2004.
(DOC)
