# Supplementary material for: Fuzziness and Heterogeneity of Benthic Metacommunities in a Complex Transitional System
Source: PLoS One. 2012 Dec 21;7(12):e52395. doi: 10.1371/journal.pone.0052395 (PMC3528783; doi:10.1371/journal.pone.0052395)
Supplement: Table S1 — Macrobenthic taxa identified on hard-bottoms in the Lagoon of Venice in 2004. (DOC) [file pone.0052395.s001.doc]

Table S1: Macrobenthic taxa identified on hard-bottoms in the Lagoon of Venice in 2004.

| **TAXON** | **CLASS** | **FREQUENCY** | **SUM (cm^2^)** | **MEAN (cm^2^)** |
| --- | --- | --- | --- | --- |
| *Acinetospora crinita* | Phaeophyta | 2 | 0.10 | 0.00 |
| *Acrochaete viridis* | Chlorophyta | 40 | 3.39 | 0.04 |
| *Aglaothamnion* sp | Rhodophyta | 32 | 157.16 | 1.96 |
| *Amphipholis squamata* | Echinodermata | 7 | 3.90 | 0.05 |
| *Amphiura chiajei* | Echinodermata | 6 | 6.50 | 0.08 |
| *Anomia ephippium* | Mollusca Bivalvia | 19 | 26.20 | 0.33 |
| *Anotrichium furcellatum* | Rhodophyta | 3 | 8.10 | 0.10 |
| Anthozoa ind | Anthozoa | 57 | 747.40 | 9.34 |
| *Antithamnion cruciatum* | Rhodophyta | 42 | 420.26 | 5.25 |
| *Antithamnion piliferum* | Rhodophyta | 7 | 111.05 | 1.39 |
| *Antithamnionella spirographidis* | Rhodophyta | 1 | 0.20 | 0.00 |
| *Ascidia mentula* | Tunicata | 1 | 0.50 | 0.01 |
| Ascidiacea ind | Tunicata | 12 | 71.40 | 0.89 |
| *Ascidiella aspersa* | Tunicata | 1 | 1.00 | 0.01 |
| *Asterina gibbosa* | Echinodermata | 9 | 38.30 | 0.48 |
| *Audouinella* sp1 | Rhodophyta | 11 | 1.42 | 0.02 |
| *Audouinella* sp2 | Rhodophyta | 47 | 12.77 | 0.16 |
| *Balanus amphitrite* | Crustacea Cirripedia | 77 | 5883.80 | 73.55 |
| *Balanus eburneus* | Crustacea Cirripedia | 64 | 6598.10 | 82.48 |
| *Balanus improvisus* | Crustacea Cirripedia | 72 | 332.95 | 4.16 |
| *Balanus perforatus* | Crustacea Cirripedia | 6 | 8.30 | 0.10 |
| *Bangia fuscopurpurea* | Rhodophyta | 58 | 6.68 | 0.08 |
| *Blidingia marginata* | Chlorophyta | 45 | 292.56 | 3.66 |
| *Blidingia minima* | Chlorophyta | 13 | 68.81 | 0.86 |
| *Botryllus schlosseri* | Tunicata | 6 | 15.70 | 0.20 |
| *Bowerbankia gracilis* | Bryozoa | 46 | 448.62 | 5.61 |
| *Bowerbankia imbricata* | Bryozoa | 1 | 0.30 | 0.00 |
| *Bryopsis plumosa* | Chlorophyta | 53 | 252.45 | 3.16 |
| *Bugula fulva* | Bryozoa | 2 | 11.00 | 0.14 |
| *Bugula neritina* | Bryozoa | 18 | 743.02 | 9.29 |
| *Bugula plumosa* | Bryozoa | 5 | 271.60 | 3.40 |
| *Bugula stolonifera* | Bryozoa | 19 | 91.25 | 1.14 |
| *Buskia socialis* | Bryozoa | 13 | 49.70 | 0.62 |
| *Callithamnion corymbosum* | Rhodophyta | 19 | 110.50 | 1.38 |
| Campanulariidae ind | Hydrozoa | 13 | 7.75 | 0.10 |
| Capitellidae ind | Polychaeta | 1 | 0.20 | 0.00 |
| *Caulacanthus ustulatus* | Rhodophyta | 1 | 0.10 | 0.00 |
| *Centroceras clavulatum* | Rhodophyta | 1 | 60.00 | 0.75 |
| *Ceramium ciliatum* | Rhodophyta | 2 | 8.20 | 0.10 |
| *Ceramium diaphanum* | Rhodophyta | 47 | 505.10 | 6.31 |
| *Chaetomorpha ligustica* | Chlorophyta | 33 | 32.78 | 0.41 |
| *Chaetomorpha linum* | Chlorophyta | 52 | 139.12 | 1.74 |
| *Chondria capillaris* | Rhodophyta | 21 | 98.93 | 1.24 |
| *Chthamalus montagui* | Crustacea Cirripedia | 6 | 8.70 | 0.11 |
| *Chthamalus stellatus* | Crustacea Cirripedia | 12 | 27.10 | 0.34 |
| *Ciona intestinalis* | Tunicata | 10 | 119.70 | 1.50 |
| *Cladophora albida* | Chlorophyta | 23 | 27.15 | 0.34 |
| *Cladophora coelothrix* | Chlorophyta | 2 | 52.00 | 0.65 |
| *Cladophora dalmatica* | Chlorophyta | 2 | 2.10 | 0.03 |
| *Cladophora hutchinsiae* | Chlorophyta | 19 | 21.40 | 0.27 |
| *Cladophora laetevirens* | Chlorophyta | 2 | 1.50 | 0.02 |
| *Cladophora rupestris* | Chlorophyta | 57 | 323.27 | 4.04 |
| *Cladophora sericea* | Chlorophyta | 58 | 170.37 | 2.13 |
| *Cladophora vagabunda* | Chlorophyta | 2 | 0.40 | 0.01 |
| *Clavelina lepadiformis* | Tunicata | 1 | 5.50 | 0.07 |
| *Codium fragile* | Chlorophyta | 7 | 12.30 | 0.15 |
| colonial Botryllidae | Tunicata | 32 | 938.40 | 11.73 |
| *Conopeum seurati* | Bryozoa | 13 | 74.55 | 0.93 |
| *Corbula gibba* | Mollusca Bivalvia | 1 | 0.05 | 0.00 |
| *Crassostrea gigas* | Mollusca Bivalvia | 71 | 26364.00 | 329.55 |
| *Cryptosula pallasiana* | Bryozoa | 7 | 144.80 | 1.81 |
| *Dasya baillouviana* | Rhodophyta | 18 | 359.63 | 4.50 |
| *Dasya hutchinsiae* | Rhodophyta | 2 | 1.40 | 0.02 |
| *Dictyota dichotoma* var. *dichotoma* | Phaeophyta | 3 | 11.00 | 0.14 |
| *Dictyota dichotoma* var. *intricata* | Phaeophyta | 2 | 302.00 | 3.78 |
| *Dictyota linearis* | Phaeophyta | 1 | 2.00 | 0.03 |
| *Dictyota* sp | Phaeophyta | 3 | 1.00 | 0.01 |
| *Didemnum maculosum* | Tunicata | 10 | 63.90 | 0.80 |
| Ectocarpales ind | Phaeophyta | 34 | 8.57 | 0.11 |
| *Ectocarpus siliculosus* var. *pygmaeus* | Phaeophyta | 7 | 1.96 | 0.02 |
| *Ectocarpus siliculosus* var. *siliculosus* | Phaeophyta | 3 | 1.04 | 0.01 |
| *Ectopleura crocea* | Hydrozoa | 8 | 360.30 | 4.50 |
| *Electra monostachys* | Bryozoa | 29 | 253.90 | 3.17 |
| *Epicladia flustrae* | Chlorophyta | 14 | 4.75 | 0.06 |
| *Erythrocladia irregularis* | Rhodophyta | 28 | 13.24 | 0.17 |
| *Eudendrium racemosum* | Hydrozoa | 2 | 27.00 | 0.34 |
| *Euraphia depressa* | Crustacea Cirripedia | 4 | 4.80 | 0.06 |
| *Ficopomatus enigmaticus* | Polychaeta | 5 | 3.60 | 0.05 |
| *Fucus virsoides* | Phaeophyta | 1 | 500.00 | 6.25 |
| *Gayralia oxysperma* | Chlorophyta | 18 | 69.08 | 0.86 |
| *Gelidium pusillum* | Rhodophyta | 10 | 150.71 | 1.88 |
| *Gonothyraea loveni* | Hydrozoa | 3 | 34.00 | 0.43 |
| *Gracilariopsis longissima* | Rhodophyta | 21 | 102.70 | 1.28 |
| *Grateloupia filicina* | Rhodophyta | 3 | 12.05 | 0.15 |
| *Grateloupia turuturu* | Rhodophyta | 3 | 30.50 | 0.38 |
| *Gymnogongrus griffithsiae* | Rhodophyta | 2 | 151.00 | 1.89 |
| *Hiatella arctica* | Mollusca Bivalvia | 45 | 115.64 | 1.45 |
| *Hincksia sandriana* | Phaeophyta | 4 | 2.30 | 0.03 |
| *Hincksia* sp | Phaeophyta | 4 | 0.75 | 0.01 |
| *Hydroides dianthus* | Polychaeta | 66 | 221.50 | 2.77 |
| *Hydroides pseudouncinatus* | Polychaeta | 1 | 0.20 | 0.00 |
| *Hydrolithon boreale* | Rhodophyta | 9 | 0.21 | 0.00 |
| *Hydrolithon* sp | Rhodophyta | 4 | 0.04 | 0.00 |
| *Hydrozoa* ind | Hydrozoa | 5 | 27.10 | 0.34 |
| *Hymeniacidon perlevis* | Porifera | 59 | 7214.00 | 90.18 |
| *Kirchenpaueria halecioides* | Hydrozoa | 48 | 319.10 | 3.99 |
| *Laurencia obtusa* | Rhodophyta | 2 | 6.50 | 0.08 |
| *Leucandra aspera* | Porifera | 1 | 1.00 | 0.01 |
| *Limnoperna securis* | Mollusca Bivalvia | 27 | 1200.80 | 15.01 |
| *Lithophyllum pustulatum* | Rhodophyta | 25 | 83.95 | 1.05 |
| *Lomentaria clavellosa* | Rhodophyta | 1 | 2.00 | 0.03 |
| *Melobesia membranacea* | Rhodophyta | 1 | 0.01 | 0.00 |
| *Modiolarca subpicta* | Mollusca Bivalvia | 32 | 40.83 | 0.51 |
| *Modiolus adriaticus* | Mollusca Bivalvia | 2 | 1.03 | 0.01 |
| *Modiolus barbatus* | Mollusca Bivalvia | 52 | 368.90 | 4.61 |
| *Modiolus* sp | Tunicata | 10 | 42.00 | 0.53 |
| *Musculista senhousia* | Mollusca Bivalvia | 15 | 11.20 | 0.14 |
| *Mycale contareni* | Porifera | 7 | 316.00 | 3.95 |
| *Myrionema orbiculare* | Phaeophyta | 7 | 34.06 | 0.43 |
| *Mytilaster lineatus* | Mollusca Bivalvia | 50 | 390.25 | 4.88 |
| *Mytilaster minimus* | Mollusca Bivalvia | 32 | 336.60 | 4.21 |
| *Mytilus galloprovincialis* | Mollusca Bivalvia | 72 | 21877.20 | 273.47 |
| *Neosiphonia harveyi* | Rhodophyta | 36 | 631.30 | 7.89 |
| *Obelia dichotoma* | Hydrozoa | 3 | 18.20 | 0.23 |
| *Ophiothrix fragilis* | Echinodermata | 7 | 54.00 | 0.68 |
| Ophiuroidea ind | Echinodermata | 6 | 4.10 | 0.05 |
| *Ostrea edulis* | Mollusca Bivalvia | 12 | 225.50 | 2.82 |
| *Petricola lithophaga* | Mollusca Bivalvia | 15 | 25.90 | 0.32 |
| *Peyssonnelia dubyi* | Rhodophyta | 9 | 23.67 | 0.30 |
| *Pneophyllum fragile* | Rhodophyta | 5 | 0.31 | 0.00 |
| Polyplacophora ind | Polyplacophora | 3 | 1.60 | 0.02 |
| *Polysiphonia denudata* | Rhodophyta | 49 | 3478.23 | 43.48 |
| *Polysiphonia elongata* | Rhodophyta | 20 | 380.52 | 4.76 |
| *Polysiphonia fibrillosa* | Rhodophyta | 7 | 79.90 | 1.00 |
| *Polysiphonia morrowii* | Rhodophyta | 10 | 194.70 | 2.43 |
| *Polysiphonia scopulorum* | Rhodophyta | 62 | 1266.70 | 15.83 |
| *Polysiphonia* sp1 | Rhodophyta | 3 | 52.00 | 0.65 |
| *Polysiphonia* sp2 | Rhodophyta | 1 | 1.00 | 0.01 |
| *Polysiphonia* sp3 | Rhodophyta | 1 | 0.01 | 0.00 |
| *Polysiphonia* sp4 | Rhodophyta | 1 | 30.00 | 0.38 |
| *Polysiphonia* sp5 | Rhodophyta | 2 | 0.40 | 0.01 |
| Porifera sp2 | Porifera | 23 | 472.80 | 5.91 |
| *Porphyra leucosticta* | Rhodophyta | 17 | 9.00 | 0.11 |
| *Pseudolithoderma adriaticum* | Phaeophyta | 1 | 3.50 | 0.04 |
| *Pyura microcosmus* | Tunicata | 2 | 10.00 | 0.13 |
| *Radicilingua thysanorhizans* | Rhodophyta | 8 | 52.00 | 0.65 |
| *Rhodophyllis divaricata* | Rhodophyta | 6 | 49.80 | 0.62 |
| *Rhodymenia ardissonei* | Rhodophyta | 35 | 1039.23 | 12.99 |
| *Sabella spallanzanii* | Polychaeta | 1 | 1.00 | 0.01 |
| *Sabellaria spinulosa* | Polychaeta | 3 | 0.80 | 0.01 |
| Sabellidae ind | Polychaeta | 2 | 0.50 | 0.01 |
| *Sahlingia subintegra* | Rhodophyta | 1 | 0.01 | 0.00 |
| *Schizoporella errata* | Bryozoa | 12 | 118.80 | 1.49 |
| *Scrupocellaria bertholettii* | Bryozoa | 1 | 25.00 | 0.31 |
| *Scytosiphon* sp | Phaeophyta | 1 | 0.20 | 0.00 |
| *Serpula vermicularis* | Polychaeta | 1 | 0.20 | 0.00 |
| *Spermothamnion repens* | Rhodophyta | 1 | 0.20 | 0.00 |
| *Spirobranchus triqueter* | Polychaeta | 23 | 17.20 | 0.22 |
| Spirorbidae ind | Polychaeta | 32 | 92.10 | 1.15 |
| *Spyridia filamentosa* | Rhodophyta | 1 | 0.03 | 0.00 |
| *Styela plicata* | Tunicata | 40 | 1316.10 | 16.45 |
| *Stylonema alsidii* | Rhodophyta | 38 | 4.41 | 0.06 |
| *Suberites carnosus* | Porifera | 3 | 58.50 | 0.73 |
| *Sycon raphanus* | Porifera | 9 | 3.80 | 0.05 |
| *Tedania anhelans* | Porifera | 14 | 2475.00 | 30.94 |
| *Tellamia* sp | Chlorophyta | 1 | 0.01 | 0.00 |
| Terebellidae ind | Polychaeta | 41 | 129.10 | 1.61 |
| *Tricellaria inopinata* | Bryozoa | 45 | 907.05 | 11.34 |
| tube-dwelling Amphipoda | Crustacea Amphipoda | 77 | 5700.00 | 71.25 |
| *Ulothrix flacca* | Chlorophyta | 6 | 6.13 | 0.08 |
| *Ulva clathrata* | Chlorophyta | 3 | 71.10 | 0.89 |
| *Ulva flexuosa* | Chlorophyta | 62 | 1466.80 | 18.34 |
| *Ulva intestinalis* | Chlorophyta | 76 | 5745.55 | 71.82 |
| *Ulva laetevirens* | Chlorophyta | 74 | 11129.00 | 139.11 |
| *Ulvella lens* | Chlorophyta | 14 | 9.33 | 0.12 |
| *Undaria pinnatifida* | Phaeophyta | 2 | 770.00 | 9.63 |
| *Vaucheria submarina* | Chrysophyta | 8 | 1.85 | 0.02 |
| *Vermiliopsis infundibulum* | Polychaeta | 5 | 3.60 | 0.05 |
